# Should Neoadjuvant Treatment Be Adopted More Widely for Patients With Extremity Soft Tissue Sarcoma in Low-Income Countries?

**DOI:** 10.1200/GO.23.00110

**Published:** 2023-07-13

**Authors:** Ankit Mangla

**Affiliations:** Ankit Mangla, MD, University Hospitals Seidman Cancer Center, Cleveland, OH, Case Western Reserve University School of Medicine, Cleveland, OH, Case Comprehensive Cancer Center, Cleveland, OH

## TO THE EDITOR:

The article by Raza et al^1^ regarding the management of localized extremity soft tissue sarcoma (ESTS) in a tertiary care center in Pakistan is a valuable insight into how these rare tumors are treated in low-income countries. Surgical resection with perioperative radiation therapy (RT) is the standard of care for patients presenting with localized ESTS.^2^ The use of neoadjuvant or adjuvant chemotherapy in patients with localized high-risk soft tissue sarcoma (STS) is an ongoing debate. However, nomograms from the Sloan-Kettering center and SARCULATOR have been validated in retrospective cohorts to help identify patients who may benefit from neoadjuvant chemotherapy.^3^ The authors report 72% of patients had positive margins after surgical resection (it is unclear if this was microscopic or macroscopic positive margin). In addition, the overall survival (OS) and disease-free survival (DFS) at 2 years are 50% and 63%, respectively, with a median DFS of 8 months. Although they cite the advanced stage as a reason for such numbers, perhaps adopting neoadjuvant therapies (both radiation and chemotherapy) for such patients may help truncate these numbers, even in patients who present with stage T2 and above ESTS.

Both neoadjuvant RT and adjuvant RT are optimal for patients with localized ESTS with similar local control rate reported in randomized studies.^4^ Neoadjuvant radiation therapy (NART) has several advantages in patients with localized ESTS. It allows the radiation oncologist to target a defined area of tumor, which results in using a lower dose of radiation (approximately 50 Gy) and smaller volumes.^4^ In comparison, patients receiving adjuvant radiation receive a higher dose (approximately 66 Gy) to a large area, including the surgical margin, which increases the risk of long-term complications. Although the risk of major wound complications (MWC) after surgery is higher with NART, these are reversible.^4^ In addition, the risk of MWC is higher with lower-extremity STS compared with upper-extremity STS, which can be mitigated with flap reconstruction. In contrast, the patients who receive adjuvant radiation usually suffer from irreversible adverse effects like lymphedema, fibrosis, decreased range of motion, and bone fracture, which are a direct result of using a higher dose of RT and covering a larger area.^4^ It would be worth exploring the long-term toxicity of adjuvant RT in the cohort presented here to develop the best practice for such patients in low-income countries. The authors report 72% of patients with positive margins, which is likely due to upfront surgery offered to patients with large tumors (T2 and above). Herein, adopting a neoadjuvant approach may help facilitate a higher rate of negative margins on microscopy (R0 resection) as NART induces reduced vascularity within the tumor leading to necrosis and improves tumor definition, which facilitates surgical resection.^4^

The authors report a 33% local recurrence rate (LRR), which they attribute to no use of chemotherapy in the neoadjuvant or adjuvant setting.^1^ On the contrary, we suspect that the high LRR could be attributed to positive margins, which is the most significant risk factor for increased local recurrence.^5^ Neoadjuvant chemotherapy (three cycles of epirubicin and ifosfamide) followed by NART can potentially offset the ill effects of close margins as shown in the randomized trial from the Italian and Spanish Sarcoma groups.^6^ The authors also report an OS of 50%, a DFS rate of 63%, and a median DFS of 8 months. Neoadjuvant chemotherapy can afford survival benefits in a select group of patients. Judicious use of widely available Sloan-Kettering nomogram or other nomograms like SARCULATOR can help identify patients who can benefit from neoadjuvant chemotherapy. The post hoc analysis of the ISG-STS 1001 trial demonstrated a survival benefit for patients receiving three cycles of epirubicin-ifosfamide in patients deemed to be high-risk per the SARCULATOR nomogram.^7^ In the neoadjuvant setting, three cycles of anthracycline-ifosfamide is the most favored regimen for patients with high-grade, deep-seated ESTS, which is larger than 5 cm.^8^

The cohort presented in this manuscript needs to be explored further as it is a significant opportunity to identify barriers to NART in patients with localized ESTS in low-income countries. In addition, perhaps a nomogram-based scoring of reported patients will help identify patients who could have benefited from neoadjuvant chemotherapy. Overall, through this cohort of patients, the authors can potentially identify significant gaps in delivering optimal care to these patients and propose a policy change at a national level to help organize the standard-of-care practice for patients with localized ESTS in low-income countries.
